# A hybrid of Bees algorithm and regulatory on/off minimization for optimizing lactate and succinate production

**DOI:** 10.1515/jib-2022-0003

**Published:** 2022-07-19

**Authors:** Mohd Izzat Yong, Mohd Saberi Mohamad, Yee Wen Choon, Weng Howe Chan, Hasyiya Karimah Adli, Khairul Nizar Syazwan WSW, Nooraini Yusoff, Muhammad Akmal Remli

**Affiliations:** Artificial Intelligence and Bioinformatics Research Group, Faculty of Computing, Universiti Teknologi Malaysia, 81310 Johor, Malaysia; Health Data Science Lab Department of Genetics and Genomics,College of Medical and Health Sciences, United Arab Emirates University, P.O. Box 17666, Al Ain, Abu Dhabi, United Arab Emirates; Big Data Analytics Center, United Arab Emirates University, Al Ain, Abu Dhabi, United Arab Emirates; Institute for Artificial Intelligence and Big Data, Universiti Malaysia Kelantan, Kota Bharu, 16100, Kelantan, Malaysia; Department of Data Science, Universiti Malaysia Kelantan, City Campus, Pengkalan Chepa, 16100 Kota Bharu, Kelantan, Malaysia,

**Keywords:** artificial intelligence, bioinformatics, data science, metabolic engineering, modelling, optimization

## Abstract

Metabolic engineering has expanded in importance and employment in recent years and is now extensively applied particularly in the production of biomass from microbes. Metabolic network models have been employed extravagantly in computational processes developed to enhance metabolic production and suggest changes in organisms. The crucial issue has been the unrealistic flux distribution presented in prior work on rational modelling framework adopting Optknock and OptGene. In order to address the problem, a hybrid of Bees Algorithm and Regulatory On/Off Minimization (BAROOM) is used. By employing *Escherichia coli* as the model organism, the most excellent set of genes in *E. coli* that can be removed and advance the production of succinate can be decided. Evidences shows that BAROOM outperforms alternative strategies used to escalate in succinate production in model organisms like *E. coli* by selecting the best set of genes to be removed.

## 1 Introduction

Gene knockout strategy is a typical genetic engineering method for deleting genes in organisms in order to understand their consequences. Several genes can be knocked out at the same time, for example, a double or triple knockout, which inactivates two or three genes together.

The *Escherichia coli* (*E. coli*) K-12 is a well-studied mutant strain that can be adopted in investigating unknown gene functions and gene regulatory networks utilising the gene knockout method. It has also been employed in examining mutational effects in the parent strain *E. coli* K-12 BW25113 [1].

Even though OptKnock and OptGene have previously substantiated their capabilities to determine knockout genes that boost metabolite production, their durability in global, local, multivariable, and multimodal function optimization is lacking. As a result, new methods are compulsory to overcome these obstacles.

Utilising search algorithms, researchers target near-perfect solutions in a fair amount of time. One of the search algorithms, swarm-based algorithm, has the ability of finding favourable answers. Swarm-based algorithms, unlike direct search algorithms, use a population of results rather than using one answer for every repetition. Bees Algorithm (BA) [2] is a Swarm-based algorithm that adequately searches for preferable solutions when contrasted to past algorithms by emulating food seeking honeybees. In BA, optimization problems can be overcome by using a mix of neighbourhood and random search. BA is widely used in combinatorial and functional optimization. Despite the fact that there is evidence that show BA is effective in overcoming optimization issues like controller formation, image analysis and multi-objective optimization which employs meta-heuristic optimization, BA’s random search dependency has rendered it to be ineffective in local search activities.

[3] proved that the combination of BA and FBA, i.e. BAFBA is more sophisticated in anticipating the preferable gene to be removed to advance the growth and output yield. BAFBA’s capacity to locate the most promising solutions and to selectively seek for the global maximum of the objective function by exploring the neighbourhoods is credited with the enhanced performance. Other hybrids, such as the Bees Hill Flux Balance Analysis, which includes the Hill climbing algorithm further showed the potential of BA to elaborate performance when hybridised [4].

Regulatory On/Off Maximisation (ROOM) is an appealing option for the building of a new BA hybrid. Prior existing work has proven that ROOM is more effective than FBA and MOMA when forecasting flux of ultimate metabolic steady state [5]. ROOM contrasts from MOMA in that it (1) dwindles the total number of indicative flux alterations in the wild type strain, (2) scrutinises for a flux distribution that meets the stoichiometric constraints (mass balance), as well as the thermodynamic and flux capacity constraints of a mutant, and (3) correlates better with experimental data than FBA and MOMA predicted. As a consequence, BAROOM is recommended to find gene knockout to promote the production of desired chemicals.

## 2 A hybrid of Bees algorithm and regulatory on/off minimization

**Figure 1:**
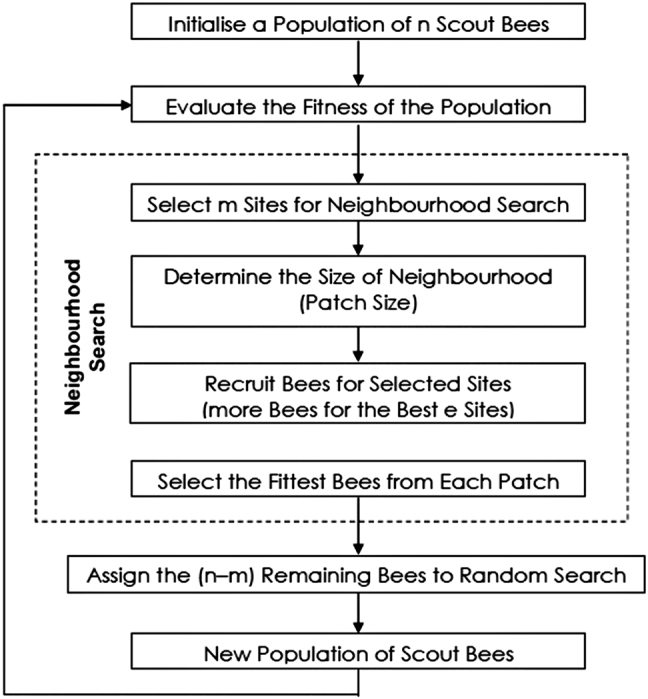
Flowchart of a basic Bees algorithm.

This paper proposes BAROOM as a strategy to analyze and decide the list of knockout. Figure 1 depicts the flow of conventional BA and, Figure 3 presents the flowchart of BAROOM. The consecutive subsections specify the details of flow.

### 2.1 Bee representation of metabolic genotype

The food foraging activity of bees commences with the scout bees being directed to promising sites. During the hunt, scout bees roam about at random. When the search is completed, the scout bees will return to the hive. When scout bees locate a site that is rated above a certain threshold, they begin to perform a “waggle dance”. This dance is made up of three pieces of information: (a) the direction in which it is located, (b) the distance from the hive, and (c) the quality of the dancing. The data assists the colony in determining the food’s quality and the quantity of energy required to harvest it. Consecutively, additional bees are dispatched directly to the most promising site together.

### 2.2 Initialization of the population

**Figure 2:**
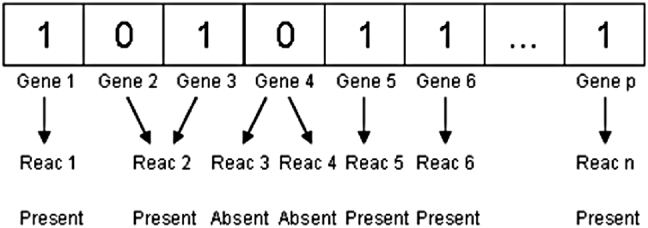
Bee representation of a metabolic genotype.

The algorithm starts with the *n* scout bees being positioned haphazardly in the search space. The first phase randomly initialises the population by stating the existence or absence of reaction as the following phase’s input. First, parameters must be set, as this is a requirement in BA. For example, the dimensions of popular, scout bees, and sites picked out of visited sites are specified as dimension, *n*, and *m*, respectively, and are set to 2000, 30, and 20. The starting size of the patch, the number of bees recruited for best e sites, the number of bees recruited for other m-e selected sites, and the number of bees recruited for other m-e selected sites are declared as e, nep, nsp, ngh, and are set to 2, 4, 8, 1 accordingly. The population is then initialised using the length of the model’s reaction list, which is 1532 reactions. The algorithm generates a random population with a matrix of 1532 (number of reactions) × 2000 (dimension of the matrix) as the dimension of the matrix must be higher than the number of reactions. As a result, a population with a matrix of 1532 × 2000 dimensions is produced at random, with the values of 0 and 1 representing the absence and existence of reactions, respectively. The metabolic genotype is represented by a bee in Figure 2.

**Figure 3:**
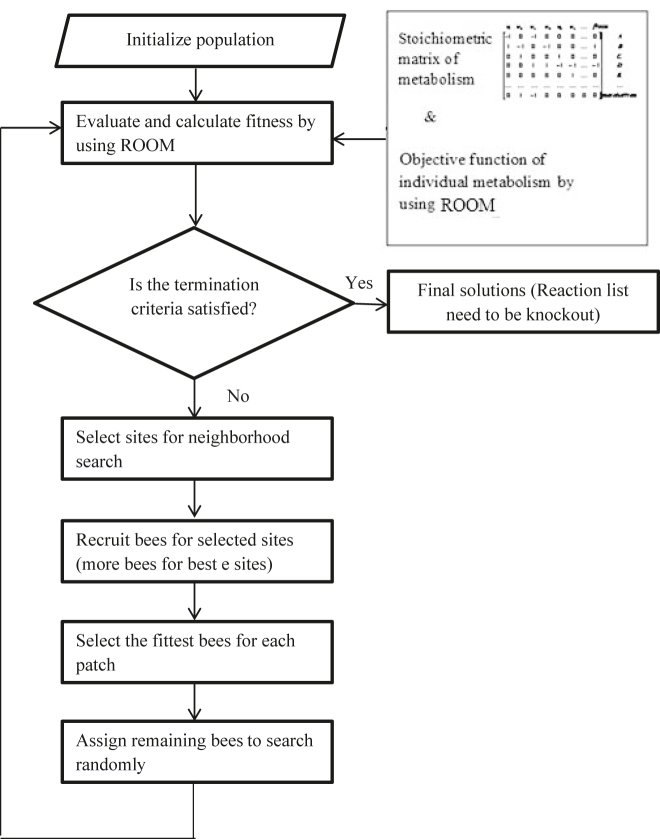
The flow chart of hybrid algorithm of BAROOM.

### 2.3 Scoring fitness of individuals

Using ROOM, the fitness of each single site visited by a bee is calculated. Starting with the first scout bee, which is taken and taught with the data, the remainder of scout bees are collected and trained with the data until all of the scout bees are evaluated. The conclusions will then be saved in an array and rearranged with the highest value first. After a gene is knocked out, ROOM utilises quadratic programming to hunt for a point in flux space that is nearer to the wild type point. According to Shlomi et al. [6], ROOM adopts mixed integer linear programming (MILP) to solve the following equation, where [*w* ^*l*^, *w* ^*u*^] is the substantial flux change threshold around the vector *w*:

(2.1) $$\min\overset{m}{\underset{i=1}{\sum }}y_{i}$$

Subjectto:S⋅v=0

$$v-y\left(v_{\max}-w^{u}\right)\leq w^{u}$$

(2.2) $$v-y\left(v_{\min}-w^{l}\right)\geq w^{l}$$

(2.3) $$v_{j}=0,j\in A,y_{i}\in \left\{0,1\right\}$$

$$w^{u}=w+\delta \left|w\right|+\mathrm{>\varepsilon},\;w^{i}=w-\delta \left|w\right|-\mathrm{>\varepsilon}$$

For every flux, where *i*, 1 ≤ *i* ≤ *m*, *y*_*i*_ = 1 for a substantial flux change in *v*_*i*_, *y*_*i*_ = 0. Therefore, when *y*_*i*_ = 1, inequality (2.2) and (2.3) do not impose new restrictions on *v*_*i*_ and when *y*_*i*_ = 0, inequality (2.2) and (2.3) constraint *v*_*i*_ to range declared previously. The size of *δ* and *ɛ* affects the time of running of MILP solver.

The growth rate and the minimum production of the biochemical will be returned after the calculation. The growth rate is to evaluate the survival of the cell after knocking out the gene where the growth rate must be more than 0.1 h^−1^, and the minimum production is taken into account where it must be more than −1 e^−3^mmol gDW^−1^ hr^−1^ to avoid considering the insignificant improvement.

After the computation, the biochemical’s growth rate and minimal production will be returned. The minimum production is taken into consideration where it must be higher than −1 e^−3^mmol gDW^−1^ hr^−1^ to prevent evaluating the small improvement, and the growth rate is used to measure the cell’s survival after knocking out the gene, where the growth rate must be more than 0.1 h^−1^.

### 2.4 Neighbourhood search

The following procedures are engaged in this neighbourhood/local search phase are (a) determining sites with maximal fitness by sorting fitness values in descending order and positions of population, (b) recruiting bees to fittest sites whose fitness are then evaluated by ROOM. Fitness sites with low-value are taken out of consideration. Bees are also recruited depending on their fitness, which is followed as they look for the ideal places for the prospective solution, (c) bees with high-value fitness are chosen as the new population for the next iteration. The *m* sites will be picked carelessly from *n*. Then from among *m*, the most outstanding *e* site is chosen haphazardly. More bees will be recruited to the most outstanding *e* site as it symbolizes a more favourable result.

### 2.5 Randomly assigned and termination

When local search yields no results, the leftover bees are given the task of exploring the search space at random in order to find new solutions. The fitness is recalculated, and the fixed value of lowest production is contrasted once again. The best production rate is then determined by sorting the production rate according to the values. The new population’s fitness value is determined by ROOM, which is followed by a neighbourhood search phase, random assignment, and termination phase. The algorithm iterates until the imax (maximum iteration) reaches 50, which is the termination condition. At the end of each repetition, the colony has two portions to its new population: representatives from each selected patch and other scout bees allocated to random searches.

In this paper, the selected knockout list by BAROOM is further evaluated using OptKnock [7]. If the difference between the production rate obtained from BAROOM and OptKnock is less than 0.001, the list is considered as valid solution. This saves time for the biologists because they can just consider the valid solution while carrying out their laboratory experiments. Following validation, the majority of the knockout genes have been shown in the literature to be associated to improving the targeted products. The given overproduction results are achieved by guaranteeing that a drain towards growth resources (i.e., carbon, redox potential, and energy) is accompanied by the production of a desired product due to stoichiometry.

## 3 Experimental results

This paper adopts *E. coli i*AF1260 model [8] as the dataset for the experiment. The *E. coli* model consists of 1261 genes, 2382 unique biochemical reactions, and 1668 metabolites. All simulations were performed for aerobic minimal media conditions. The glucose uptake rate was fixed to 10 mmol/gDW/hr while a set non-growth associated maintenance of 7.6 mmol ATP/gDW/hr.

### 3.1 Experimental result and discussion for succinate

3 sets of knockout lists are concluded. Table 1 demonstrates three knockout lists that are approximated by BAROOM for the production of succinate. The elimination of *tkt2* gene (precisely *TktB*), *pdh* genes (namingly *AceEec*, *AceFec* and *LpdA*) and *fum* genes (particularly *FumA*, *FumB* and *FumCec*). The removal causes 7.9633 mmol gDW^−1^ hr^−1^ succinate to be produced with the growth rate being 0.2588 (h^−1^). *tkt2* gene that is concerned in the pentose-phosphate shunt that can yield glycolytic intermediates (fructose-6-phosphate (*F6P)* and glyceraldehyde-3-phosphate(*G3P)*) as the gene is to produce transketolase enzyme to speed up the reaction of the erythrose 4-posphate (*E4P*) and xylulose 5-phosphate (*XU5-PD*). According to Yanase et al. [9], it ultimately causes the precursors of succinate, which are *G3P* and *F6P* to produce less succinate, and build-up of *XU5-PD* as well inhibits the organism’s development. As a consequence, the deletion of the *tkt2* gene boosts *G3P* and *F6P* production. Withal, the elimination of *pdh* genes draws the impact of the pyruvate (PYR) being unable to change into acetyl-coA (ACCOA) by pyruvate dehydrogenase and reduces the formation of competing products such as acetate and ethanol, and it favours the conversion of phosphoenolpyruvate (PEP) into oxaloacetate (OAA) in citric acid cycle under anaerobic condition. The synthesis of succinate is boosted as OAA accumulates. Furthermore, the absence of the fumarase enzyme due to the deletion of fum genes leads to an escalation in fumarate synthesis, which may subsequently be converted to succinate by fumarase reductase [10]. As a result of the increased amount of OAA and fumarate, succinate synthesis increases considerably.

**Table 1:** List of gene knockout that identified by BAROOM for succinate production.

No.	Knockout	Enzyme	Associated gene	Succinate (mmol gDW^−1^ hr^−1^)	Growth Rate (h^−1^)
1.	E4P + XU5P-D <==> F6P + G3P COA + NAD + PYR -> ACCOA + CO2+NADH FUM + H2O<==>MAL-L	Transketolase Pyruvate dehydrogenase Fumarase	TktB AceEec and AceFec and LpdA FumA and FumB and FumCec	7.9633	0.2588
2.	6PGL + H2O->6PGC + H COA + NAD + PYR -> ACCOA + CO2+NADH Q8+SUCC -> FUM +Q8H2	6-Phosphogluconolactonase Pyruvate dehydrogenase Succinate dehydrogenase	Pgl AceEec and AceFec and LpdA Sdh	8.0942	0.2126
**3.**	**G6P + NADP <==> 6PGL +H + NADPH** **MAL-L +NAD <==> H + NADH+ OAA** **COA + NAD + PYR ->** **ACCOA + CO2+NADH** **Q8+SUCC -> FUM +Q8H2**	**Glucose 6-phosphate dehydrogenase** **Malate dehydrogenase** **Pyruvate dehydrogenase** **Succinate dehydrogenase**	**Zwf** **Mdh** **AceEec and AceFec and LpdA** **Sdh**	**8.1943**	**0.2226**

Bold font represents the best result.

Next, the cancellation of *Pgl* gene, *pdh* genes (which are *AceEec*, *AceFec* and *LpdA*) and *Sdh* gene consequences to 8.0942 mmol gDW^−1^ hr^−1^ of succinate production and 0.2126 (h^−1^) of growth rate. The knockout of *Pgl* gene, which encodes enzyme 6-phosphocluconolactonase, affects the hydrolysis of 6-phosphogluconolactone (*6PGL*) into 6-phosphogluconate (*6PGC*), according to Terzer et al. [11]. The conversion of 6PGL into glucose-6-phosphate (G6P) increases the formation of the precursor of succinate, G6P, which leads to an increase in succinate. Furthermore, eliminating Pgl prevents the secretion of competing products in the pentose phosphate pathway.

The following list is associated with the deletion of *Zwf* gene, *Mdh* gene, *pdh* genes, and *Sdh* gene that contributes to the maximal production of succinate with 8.1943 mmol gDW^−1^ hr^−1^ and 0.2226 (h^−1^) of growth rate. First, the *Zwf* gene that catalyzes the oxidation of glucose 6-phosphate (*G6P*) to 6-phosphogluconolactone (*6PGL*). This is the first reaction that enters the pentose phosphate pathway from glycolysis. As a result of the deletion of the *Zwf* gene, which changes the pentose phosphate pathway, the activity of the citric acid cycle is boosted [12]. The competing products such as ribulose 5-phosphate and ribose 5-phosphate are not secreted. The *Mdh* gene is then deleted, which catalyzes the reversible oxidation of malate (MAL-L) to oxaloacetate (OAA) [13]. As a result, OAA can be transformed into citrate, isocitrate, 2-ketoglutarate, succinyl-coA, and succinate.

#### 3.1.1 Comparative analysis for succinate case

Table 2 illustrates knockout lists that are concluded by OptKnock. As evidenced in Table 2.3, OptKnock attained the best succinate production with 6.21 mmol gDW^−1^ hr^−1^ in third knockouts, which is lower than the succinate production rate with 8.1943 mmol gDW^−1^ hr^−1^ that is identified by BAROOM. Conclusively, statistics predicted by BAROOM are better in terms of succinate production.

**Table 2:** List of gene knockouts that are identified by OptKnock for succinate production [14].

No.	Knockout	Enzyme	Succinate (mmol gDW^−1^ hr^−1^)
1.	COA +PYR -> ACCOA+ FOR NADH + PYR <==>LAC + NAD	Pyruvate formate lyase Lactate dehydrogenase	1.65
2.	COA +PYR -> ACCOA+ FOR NADH + PYR <==> LAC + NAD ACCOA+2NADH <==> COA + ETH+2NAD	Pyruvate formate lyase Lactate dehydrogenase Acetaldehyde dehydrogenase	4.79
**3.**	**ADP + PEP -> ATP + PYR** **ACTP + ADP <==> AC + ATP or ACCOA + Pi <==> ACTP + COA** **GLC + PEP -> G6P + PYR**	**Pyruvate kinase** **Acetate kinase or phosphotransacetylase** **Phosphotransferase system**	**6.21**

Bold font represents the best result.

Following that, Ren et al. [14] presented MOMAKnock as a framework for identifying genes that lead to maximizing of the desired metabolite production under the ROOM assumption. Because this article uses the same dataset, the iAF1260 model, the MOMAKnock findings in Table 3 are based on iAF1260 (*E. coli* core network). MOMAKnock determines the lists for succinate production, as shown in Table 3.7. In Table 3.7, MOMAKnock has a production of 5.02 mmol gDW^−1^ hr^−1^, which is lower than BAROOM’s production of 8.1943 mmol gDW^−1^ hr^−1^.

**Table 3:** List of gene knockout that identified by MOMAKnock for succinate production.

No.	Knockout	Succinate (mmol gDW^−1^ hr^−1^)
1.	Q8+SUCC -> FUM + Q8H2 6PGL + H2O -> 6PGC +H (2)H2O + O2 + URATE -> ALTN + CO2 + H2O2	5.02
2.	Q8+SUCC -> FUM + Q8H2 AC + ATP -> ACTP + ADP H2O + METHF -> 10FTHF R5P + XU-5P -> G3P +S7P	5.02
3.	Q8+SUCC -> FUM + Q8H2 GLU-L + H -> 4ABUT + CO2 3PG +NAD -> 3PHP +H+ NADH 3PHP + GLU-L ->AKG + PSER-L 6PGC + NADP -> CO2 + NADPH +RU5P-D	5.02

The results are also contrasted with the results from three wet laboratory tests for the synthesis of succinate. Succinate production produced from the modified *E. coli* strains is demonstrated in Table 4. According to Table 4, the maximum succinate production that is identified by Yang et al. [15] is 1.16 mmol gDW^−1^ hr^−1^ whereas the highest succinate production identified by Zhu and Shimizu [16] is 1.54 ± 0.23 mmol gDW^−1^ hr^−1^. When contrasted to the results obtained from wet laboratory experiments, BAROOM yields 8.1943 mmol gDW^−1^ hr^−1^ of succinate [15–17]. On the other hand, Jantama [17] finds the maximum succinate production with 11.78 ± 0.16 mmol gDW^−1^ hr^−1^.

**Table 4:** Succinate production for the engineered strain in *E. coli*.

No.	Reference	Relevant deletions	Succinate (mmol gDW^−1^ hr^−1^)
1.	BAROOM	Zwf,Mdh,AceEec and AceFec and LpdA,Sdh	8.1943
2.	[15]	Nuo	0.50
		ackA-pta	0.50
		**ackA-pta-nuo**	**1.16**
3.	[16]	pta	0.77 ± 0.10
		ppc	0.00 ± 0.03
		adhE	0.07 ± 0.02
		**pykF**	**1.54** ± **0.23**
4.	[17]	ldhA, adhE, ackA, pflB, mgsA, poxB, ptsG	11.49 ± 0.17
		ldhA, adhE, ackA, pflB, mgsA, poxB, galP	3.43 ± 0.10
		**ldhA, adhE, ackA, pflB, mgsA, poxB, manX**	**11.78** ± **0.16**
		ldhA, adhE, ackA, pflB, mgsA, poxB, galP, manX	2.00 ± 0.37
		ldhA, adhE, ackA, pflB, mgsA, poxB, galP, ptsG	2.24 ± 0.06

Bold font represent the best result.

#### 3.1.2 Performance measurement for succinate case

The standard deviation of the growth rate in the succinate case study are presented in Table 1. The general standard deviation of growth rate is less than 0.09, as per Table 5. Due to the low standard deviation, the growth rate in as aggregate of 50 runs is close the mean value.

**Table 5:** Performance measurement of succinate production with maximum knockout from value 1 until value 5. All values are calculated over 50 runs for each maximum knockout.

Max knockout measurement	KO = 1	KO = 2	KO = 3	KO = 4	KO = 5
**Mean (growth rate)**	0.574478455	0.429597287	0.404764112	0.395336799	0.384006433
**Standard deviation (growth rate)**	0	0.054324112	0.04320476	0.061362123	0.090804587
**Accuracy (optimal solution)**	50/50 × 100% = 100%	50/50 × 100% = 100%	50/50 × 100% = 100%	50/50 × 100% = 100%	50/50 × 100% = 100%
**Accuracy (valid solution)**	50/50 × 100% = 100%	50/50 × 100% = 100%	50/50 × 100% = 100%	50/50 × 100% = 100%	50/50 × 100% = 100%

Furthermore, BAROOM examines the final knockout list in each run and delivers the solver status for the final knockout list in a consistent format. In this study, value 1 is returned for all 50 runs, indicating the best solution. In the succinate case study, this results in 100 percent accuracy for optimal solution in all runs. The accuracy of the results is shown in Table 5.

BAROOM additionally returns the kind of solution after evaluating the final knockout list. BAROOM yields a result of 1 in this paper, indicating a correct solution in all 50 runs. As a result, all of the mutants in this research with multiple genes removed are capable of producing succinate.

## 4 Conclusion and future works

Ultimately, ultilizing the *E. coli* model, this research discusses the prediction of gene knockdown techniques given by BAROOM that lead to succinate overproduction. It implies that BAROOM can outperform earlier in silico and *in vivo* studies. This document also includes information on the BAROOM’s overall performance. Evidence has proven BAROOM to have a high level of stability and dependability, as well as the capacity to find the best and most valid solution for the final knockout list in each run. Nonetheles, there are potential further evaluations that are possible to be carried out in future, for example (a) employing different dataset and target metabolites such as *Clostridium acetobutylicum* for biofuels production [18], (b) enhancing the ability of performing local search in BA, and (c) usage of a web-server for the strategy brought up in this paper.

## References

[1] Baba T, Ara T, Hasegawa M, Takai Y, Okumura Y, Baba M (2006). Construction of Escherichia coli K-12 in-frame, single-gene knockout mutants: Keio collection. Mol Syst Biol.

[2] Pham DT, Ghanbarzadeh A, Koç E, Otri S, Rahim S, Zaidi M (2006). The bees algorithm—a novel tool for complex optimisation problems. Intelligent production machines and systems.

[3] Choon YW, Mohamad MS, Deris S, Chong CK, Chai LE, Ibrahim Z (2012). Identifying gene knockout strategies using a hybrid of bees algorithm and flux balance analysis for in silico optimization of microbial strains. Distributed computing and artificial intelligence.

[4] Choon YW, Mohamad MS, Deris S, Chong CK, Omatu S, Corchado JM (2015). Gene knockout identification using an extension of bees hill flux balance analysis.

[5] Kleessen S, Nikoloski Z (2012). Dynamic regulatory on/off minimization for biological systems under internal temporal perturbations. BMC Syst Biol.

[6] Shlomi T, Berkman O, Ruppin E (2005). Regulatory on-off minimization of metabolic flux changes after genetic perturbations. Proc Natl Acad Sci USA.

[7] Burgard AP, Pharkya P, Maranas CD (2003). OptKnock: a bilevel programming framework for identifying gene knockout strategies for microbial strain optimization. Biotechnol Bioeng.

[8] Feist AM, Zielinski DC, Orth JD, Schellenberger J, Herrgard MJ, Palsson B (2010). Model-driven evaluation of the production potential for growth coupled products of Escherichia coli. Metab Eng.

[9] Yanase H, Sato D, Yamamoto K, Matsuda S, Yamamoto S, Okamoto K (2007). Genetic engineering of zymobacter palmae for production of ethanol from xylose. Appl Environ Microbiol.

[10] Cheng K, Wang G, Zeng J, Zhang J (2013). Improved succinate production by metabolic engineering. BioMed Res Int.

[11] Terzer M, Maynard ND, Covert MW, Stelling J (2009). Genome scale metabolic networks. System Biology and Medicine.

[12] Zhao J, Baba T, Mori H, Shimizu K (2004). Effect of zwf gene knockout on the metabolism of Escherichia coli grown on glucose or acetate. Metab Eng.

[13] Park S, Cotter P, Gunsalus RP (1995). Regulation of malate dehydrogenase (mdh) gene expression in Escherichia coli in response to oxygen, carbon, and heme availability. J Bacteriol.

[14] Ren S, Zeng B, Qian X (2013). Adaptive Bi-level programming for optimal gene knockouts for targeted overproduction under phenotypic constraints. BMC Bioinf.

[15] Yang Y, Benett GN, San K (1999). Effect of inactivation of nuo and ackA-pta on redistribution of metabolic fluxes in Escherichia coli. Biotechnol Bioeng.

[16] Zhu J, Shimizu K (2005). Effect of a single-gene knockout on the metabolic regulation in Escherichia coli for D-lactate production under microaerobic condition. Metab Eng.

[17] Jantama K (2010). Glucose is taken up by galactose permease in metabolic engineered Escherichia coli to produce succinate. Suranaree J Sci Technol.

[18] Yoo M, Soucaille P (2020). Trends in systems biology for the analysis and engineering of Clostridium acetobutylicum metabolism. Trends Microbiol.

